# Investigation of Cuprizone-Induced Demyelination in mGFAP-Driven Conditional Transient Receptor Potential Ankyrin 1 (TRPA1) Receptor Knockout Mice

**DOI:** 10.3390/cells9010081

**Published:** 2019-12-28

**Authors:** Gábor Kriszta, Balázs Nemes, Zoltán Sándor, Péter Ács, Sámuel Komoly, Zoltán Berente, Kata Bölcskei, Erika Pintér

**Affiliations:** 1 Department of Pharmacology and Pharmacotherapy, University of Pécs Medical School, Pécs H-7624, Hungary; gabor.kriszta@aok.pte.hu (G.K.); balazs.nemes@aok.pte.hu (B.N.); zoltan.sandor@aok.pte.hu (Z.S.); kata.bolcskei@aok.pte.hu (K.B.); 2 Molecular Pharmacology Research Group and Center for Neuroscience, János Szentágothai Research Center, University of Pécs, Pécs H-7624, Hungary; 3 Research Group for Experimental Diagnostic Imaging, University of Pécs Medical School, Pécs H-7624, Hungary; zoltan.berente@aok.pte.hu; 4 Department of Neurology, University of Pécs Medical School, Pécs H-7623, Hungary; acs.peter@pte.hu (P.Á.); komoly.samuel@pte.hu (S.K.); 5 Department of Biochemistry and Medical Chemistry, University of Pécs Medical School, Pécs H-7624, Hungary

**Keywords:** transient receptor potential ankyrin 1, cuprizone, demyelination, astrocyte, conditional knockout, magnetic resonance imaging

## Abstract

Transient receptor potential ankyrin 1 (TRPA1) receptors are non-selective cation channels responsive to a variety of exogenous irritants and endogenous stimuli including products of oxidative stress. It is mainly expressed by primary sensory neurons; however, expression of TRPA1 by astrocytes and oligodendrocytes has recently been detected in the mouse brain. Genetic deletion of TRPA1 was shown to attenuate cuprizone-induced oligodendrocyte apoptosis and myelin loss in mice. In the present study we aimed at investigating mGFAP-Cre conditional TRPA1 knockout mice in the cuprizone model. These animals were generated by crossbreeding GFAP-Cre^+/−^ and floxed TRPA1 (TRPA1^Fl/Fl^) mice. Cuprizone was administered for 6 weeks and demyelination was followed by magnetic resonance imaging (MRI). At the end of the treatment, demyelination and glial activation was also investigated by histological methods. The results of the MRI showed that demyelination was milder at weeks 3 and 4 in both homozygous (GFAP-Cre^+/−^ TRPA1^Fl/Fl^) and heterozygous (GFAP-Cre^+/−^ TRPA1^Fl/−^) conditional knockout animals compared to Cre^−/−^ control mice. However, by week 6 of the treatment the difference was not detectable by either MRI or histological methods. In conclusion, TRPA1 receptors on astrocytes may transiently contribute to the demyelination induced by cuprizone, however, expression and function of TRPA1 receptors by other cells in the brain (oligodendrocytes, microglia, neurons) warrant further investigation.

## 1. Introduction

Transient receptor potential ankyrin 1 (TRPA1) receptors are non-selective cation channels which are responsive to a variety of exogenous and endogenous stimuli including mustard oil, cinnamaldehyde, irritant chemicals such as formalin or acrolein, as well as reactive oxygen species and oxidized lipid molecules [1,2,3]. Apart from being a nocisensor for exogenous irritant compounds, it has been suggested to work as a sensor for oxidative stress [4]. Originally described to be localized on a subgroup of nociceptive primary afferent neurons [5,6], it was later revealed that TRPA1 is also expressed at lower levels by various non-neuronal cells including keratinocytes, endothelial cells and cells of the gastrointestinal mucosa [1,2,3,7]. More importantly, several studies have supported the presence of TRPA1 receptors in the brain on astrocytes [8,9,10], as well as oligodendrocytes [11]. A recent cell-specific transcriptome analysis of the mouse cortex revealed low level expression of TRPA1 on neurons, astrocytes, oligodendrocytes and microglia, as well [12].

In astrocytes, TRPA1 receptors were implicated in both physiological and pathophysiological processes. Astrocyte TRPA1 receptors were shown to regulate resting Ca^2+^ levels and modulate GABA-ergic inhibitory transmission by reducing GABA transport [8]. TRPA1 receptors on astrocytes were also suggested to play a role in long-term potentiation in the mouse hippocampus [9]. Since reactive astrocytes can contribute to the progression of neuroinflammation in neurodegenerative diseases [13,14], several workgroups, including ours, had started to investigate the role of TRPA1 in animal models of neurodegenerative diseases. Our previous study aimed at examining the role of TRPA1 in the cuprizone-induced demyelination model in mice. Cuprizone treatment constitutes an accepted non-immune animal model of multiple sclerosis [15,16] which produces lesions resembling type III lesions seen in patients [17]. Feeding mice with cuprizone leads to a well-reproducible demyelination of the corpus callosum as well as other subcortical and cortical brain areas by inducing oligodendrocyte apoptosis and a secondary activation of astrocytes and microglia [15,16,18,19,20,21]. We have revealed that demyelination of the corpus callosum was significantly reduced in TRPA1 receptor gene-deleted mice [22,23]. Based on our data we have assumed that TRPA1 receptors localized on astrocytes may influence the astrocyte-oligodendrocyte crosstalk. Activation of these receptors on the astrocytes increases the intracellular Ca^2+^ concentration and subsequent release of mediators. Astrocyte-derived signaling molecules may contribute to the apoptosis of oligodendrocytes by promoting the proapoptotic p38-MAPK pathway resulting in c-Jun activation [22]. We have also shown that TRPA1 deficiency did not affect the number of oligodendrocyte precursor cells (OPCs) during cuprizone treatment. In TRPA1 KO mice, there was no increase or a less pronounced increase of growth factors promoting OPC proliferation (FGF-2, IGF-1). The level of Bak mRNA, a marker of apoptosis and levels of pro-apoptotic signalling proteins were significantly lower in cuprizone-treated TRPA1 KO animals. All these data suggest that TRPA1 deficiency did not affect oligodendrocyte development but reduced the apoptosis of mature oligodendrocytes [22]. In contrast with our theory, Hamilton and coworkers assumed a direct action of TRPA1 activation on myelination. They detected the functional expression of TRPA1 on oligodendrocytes and showed that ischemia-induced demyelination was diminished in the lack of TRPA1 receptors [11]. Likewise, another group showed that in TRPA1 receptor gene-deleted mice behavioral deficits and neuroinflammation were less severe in a transgenic mouse model of Alzheimer’s disease [24]. The same group also later demonstrated that the loss of TRPA1 is associated with decreased anxiety-like behavior and improved performance in spatial memory and social discrimination tests [25].

Based on these prior data we focused on further elucidating the role of TRPA1 receptors in the cuprizone model. In the present study, the effects of cuprizone in the corpus callosum were studied in mGFAP-driven conditional TRPA1 knockout mice.

## 2. Materials and Methods

### 2.1. Ethics

The study was designed and conducted according to European legislation (Directive 2010/63/EU) and Hungarian Government regulation (40/2013., II. 14.) on the protection of animals used for scientific purposes. The project was approved by the Animal Welfare Committee of the University of Pécs and the National Scientific Ethical Committee on Animal Experimentation of Hungary and licensed by the Government Office of Baranya County (license No. BA02/2000-82/2017).

### 2.2. Animals

Animals were bred in the Animal House of the Department of Pharmacology and Pharmacotherapy of the University of Pécs and kept in the Animal House of the Szentágothai Research Center during the experiments. Mice were housed in groups of 3–7 in standard polycarbonate cages on wood shavings bedding. Food and water were provided *ad libitum*. The temperature was maintained at 24 °C and the lighting was set to a 12 h light-dark cycle (lights on from 6:00 a.m. to 6:00 p.m.).

The mGFAP-driven conditional TRPA1 receptor knockout mice were produced via crossbreeding floxed TRPA1 carrying mice (B6.129S-Trpa1tm2Kykw/J) by GFAP promoter directed Cre recombinase gene expressing mice (B6.Cg-Tg(Gfap-cre) 77.6Mvs/2J) both obtained from The Jackson Laboratory (Bar Harbor, ME, USA), stock numbers #008650 and #024098, respectively. Only female mice carrying the GFAP-Cre transgene were used to prevent non-specific Cre activation during spermatogenesis. The GFAP-Cre transgene was also maintained in heterozygote form, to minimize the chance of non-specific recombination. As the first step, GFAP-Cre heterozygote females were crossbred by floxed TRPA1 homozygote males. Next, female mice heterozygote for both genes were backcrossed by floxed TRPA1 homozygote males. The obtained male Cre^+/−^ TRPA1^Fl/Fl^ mice were the main subjects of the experiments, whereas their male siblings (Cre^+/−^ TRPA1^Fl/−^ and Cre^−/−^ TRPA1^Fl/Fl^) were used as hetero controls and Cre negative controls, respectively. Animals showing a global KO genotype by tail analysis were excluded from the experiments (approximately 10% of all genotyped Cre^+/−^ animals).

Genotyping of floxed TRPA1 was done according to the protocol suggested by the provider, using forward primer oIMR9168 (AGC AGG AGC AGA AGT ATG GAA) and reverse primer oIMR9169 (GAA GGC CAT GGC ATC TTA AC) producing 359 bp (floxed) and/or 472 bp (wild type) PCR products.

Genotyping of GFAP-Cre was done by forward primer 15831 (TCC ATA AAG GCC CTG ACA TC) and reverse primer 15832 (TGC GAA CCT CAT CAC TCG T) also using internal positive control forward primer oIMR8744 (CAA ATG TTG CTT GTC TGG TG) and reverse primer oIMR8745 (GTC AGT CGA GTG CAC AGT TT) which produced 200 bp (internal positive control) and ~400 bp (GFAP-Cre positive) PCR products. Genotyping of conditional TRPA1 receptor knockout was carried out by using forward primer exon 21 (TGT TCC TCA ACA TCC CAG CG), second forward primer oIMR9168 (AGC AGG AGC AGA AGT ATG GAA) and reverse primer oIMR9169 (GAA GGC CAT GGC ATC TTA AC) producing 609 bp (knockout), 359 bp (intact floxed) and/or 472 bp (intact wild type) PCR products, respectively. RT-PCR analysis of conditional TRPA1 receptor knockout messenger RNA was done by forward primer exon 21 (TGT TCC TCA ACA TCC CAG CG) and reverse primer exon 25 (CGT GCC TGG GTC TAT TTG GA) which produce 573 bp (intact) or 191 bp (knockout) RT-PCR products.

### 2.3. Mouse TRPA1 Quantitative RT-PCR

Total RNA was isolated from homogenized brain samples by using TRI Reagent (Molecular Research Centre Inc., Cincinnati, OH, USA) and Direct-Zol RNA isolation kit (Zymo Research, Irvine, CA, USA) according to the manufacturer’s instructions. Purified RNA was quantified by a NanoDrop ND-1000 spectrophotometer and 1 µg total RNA was treated with DNAse I (Thermo Scientific, Waltham, MA, USA) to remove genomic DNA contamination from the samples. First strand cDNA synthesis was carried out with 0.5 µg of total RNA/sample using Maxima™ First Strand cDNA Synthesis Kit for RT-qPCR (Thermo Scientific, Waltham, MA, USA). In the PCR reaction we used SensiFast Probe Lo-ROX Kit (Bioline Inc., London, UK) and forward primer 5’ atgccttcagcaccccattg (binding site in exon 23), reverse primer 5’ gacctcagcaatgtccccaa (binding site in exon 24) and labeled probe 56FAM/tgggcagct/ZEN/tattgccttcacaat/3IABkFQ (binding site in exon 23), 1 µM each, all obtained from Integrated DNA Technologies (Coralville, IA, USA). The following PCR protocol was used on an Applied Biosystems (Foster City, CA, USA) Quantstudio5 quantitative PCR machine: 3 min 95 °C original denaturation, followed by 40 cycles of 30 s 95 °C denaturation, 30 s 62 °C annealing, 1 min 72 °C extension. The sites of the primers and probe are missing from the Cre-Lox recombined mRNA therefore this assay measures only the amount of intact TRPA1 mRNA. Mouse beta-actin mRNA was used as reference and the expression was determined by using Prime Time Std qPCR Assay Mm.PT.58.33540333 obtained from Integrated DNA Technologies (Coralville, IA, USA) under the same PCR conditions. All qRT-PCR assays were done in duplicates in each sample and Ct values were determined. dCt values were obtained by subtracting the corresponding beta-actin Ct values from the TRPA1 Ct values. ddCt values were calculated for each sample by subtracting the average dCt value of the Cre^−/−^ group (representing the 100% expression level) from each dCt value. Finally, for each sample the obtained ddCt were converted to relative expression level by the 100/(2^ddCt^) formula.

### 2.4. Cuprizone Treatment

Cuprizone was administered orally for 6 weeks as previously described [22,23]. Briefly, standard rodent chow was ground and 0.2% cuprizone was thoroughly mixed into ground chow which was placed into the cages in small ceramic bowls. Control mice were fed the milled chow without the addition of cuprizone. The chow was provided *ad libitum* to all groups and it was changed to a fresh batch each day. The general health status of animals was also monitored daily and body weights were measured every 2 days.

### 2.5. Magnetic Resonance Imaging (MRI)

The timeline of demyelination was monitored by T2-weighted MRI measurements on 4 mice from each group. Animals were scanned once before cuprizone administration and later once per week starting from week 2 of the treatment. The measurements were performed using a Bruker^®^ PharmaScan^®^ (4.7 T) small animal MRI instrument (Bruker, Billerica, MA, USA).

Anesthesia was induced by 3.5% *v*/*v* of isoflurane in a gas mixture of 33% O_2_ and 66% N_2_O via induction chamber and maintained with 1–2% isoflurane in a rodent face mask controlled by respiratory monitoring and gating system.

The imaging protocol was performed on the same day and same time each week. After B_0_ mapping a multislice T2 Rapid Acquisition with Relaxation Enhancement (RARE) experiment was performed (TR/TE: 3000/50 ms, FOV: 16 × 16 mm, Thk: 0.8 mm, Gap: 0.2 mm, matrix: 160 × 160, 4 averages, RARE factor: 4) on seven coronal slices positioned to cover the whole corpus callosum. The total imaging time was roughly 12 min per animal.

Before the analysis, all imaging data were converted first into a Digital Imaging Communications in Medicines (DICOM) format and stored in an isolated hardware in a local system. Any further processing was performed via DICOM-handling software packages. (3D-Slicer v4.6 NA-MIC and Onis v2.5, Digitalcore, Tokyo, Japan) [26]. For the quantification of the damage, regions of interests (ROI) were manually circumscribed, fitted by anatomical structures in the medial corpus callosum and the signal intensities (as mean ± SEM) of ROIs have been recorded. Results were expressed in percentage of intensity ratio, compared to the pre-treatment intensity (considered as 100%) measured in the identical brain area of the same individual animal. The evaluation was performed in a blinded manner.

### 2.6. Histological Assessment of Cuprizone-Induced Changes in the Corpus Callosum

Animals were anesthetized with pentobarbital (70 mg/kg i.p.) at the end of the cuprizone treatment (week 6) and perfused transcardially in two steps, first with phosphate buffered saline (PBS, pH 7.4) and then with 4% paraformaldehyde in 0.1M phosphate buffer. Brains were postfixed over one night in the same fixative. 5 μm thin coronal sections from paraffin embedded brains were made and mounted onto a silane-coated slides.

#### 2.6.1. Luxol Fast Blue-cresyl Violet (LFB/CV) Staining

LFB/CV staining was used to evaluate the severity of demyelination as described previously [20,22] on coronal sections obtained from different regions (0.14, −0.22,−1.06, and −1.94 mm) according to the mouse brain atlas of Paxinos and Franklin [27]. Brain sections on silane-coated slides were rehydrated in graded series of alcohol and incubated at 60 °C in LFB solution (0.01%), overnight. Thereafter, sections were differentiated in a solution of Li_2_CO_3_ (0.05%) and counterstained with CV. Four sections of each animal were scored by a semiquantitative four-tiered scoring system (0–3) in a blinded manner. Intact myelin was scored with 0 and the totally damaged myelin was labeled with score 3.

#### 2.6.2. Immunohistochemical Detection of Myelin Basic Protein (MBP) and Astrocyte and Microglia Markers

In addition to the LFB/CV staining, immunohistochemical detection of myelin basic protein (MBP) was also performed to evaluate demyelination. Furthermore, astrocyte and microglia activation in the corpus callosum was detected with immunohistochemical staining of glial fibrillary acidic protein (GFAP) and ionized calcium-binding adaptor molecule 1 (Iba1), respectively. Briefly, 8 μm-thick paraffin sections were deparaffinized and heat-unmasked in citrate buffer. Sections were treated with 3% hydrogen peroxide to block endogenous peroxidase activity, treated with BSA and incubated for 1 h with the following primary antibodies: anti-MBP (1:100, mouse monoclonal antibody from Novocastra/Leica Biosystems (Nussloch, Germany, catalogue No. NCL-MBP; Antibody Registry No AB_563893), anti-GFAP (1:1000, rabbit polyclonal antibody from Dako, Glostrup, Denmark; catalogue No. Z0334; Antibody Registry No AB_10013382) to visualize astrocytes, anti-Iba1, (1:500, rabbit polyclonal antibody from Wako Chemicals, Neuss, Germany; catalogue No. 019-19741; Antibody Registry No AB_839504) as a marker for microglia/macrophages. Incubation was performed with the HISTO-Labeling System, the reaction was visualized using 3,3′-diaminobenzidine reaction. Sections were photographed with an Olympus DP50 camera attached to an Olympus BX51 microscope (Olympus, Tokyo, Japan) under 200x magnification. Quantification was performed by determining the mean density of equal-sized regions of interest of the medial corpus callosum with the Image-ProPlus software (Media Cybernetics, Rockville, MD, USA). The experimenter evaluating the slides was blinded to the group allocation.

### 2.7. Materials

Cuprizone and all other chemicals were purchased from Sigma-Aldrich (St. Louis, MO, USA), unless otherwise previously indicated. Pentobarbital (Euthanimal 20% injection ad us. vet.) was obtained from Alfasan Nederland B.V. (Woerden, The Netherlands) while isoflurane was purchased from Medicus Partner Ltd. (Biatorbágy, Hungary).

### 2.8. Statistics

In each group, means ± S.E.M. of parameters were calculated. Statistical analysis was carried out with the GraphPad Prism 8.0.1 software (GraphPad Software Inc., San Diego, CA, USA). For comparison of qPCR relative expression values and MRI relative intensity values, one-way ANOVA followed by Tukey’s multiple comparisons test was used. Paired t-test was used to compare density values of LFB/CV and immunohistological staining, *p < 0.05 or **p < 0.01 was considered as statistically significant.

## 3. Results

### 3.1. Determination of TRPA1 mRNA Levels in the Mouse Brain

**Figure 1:**
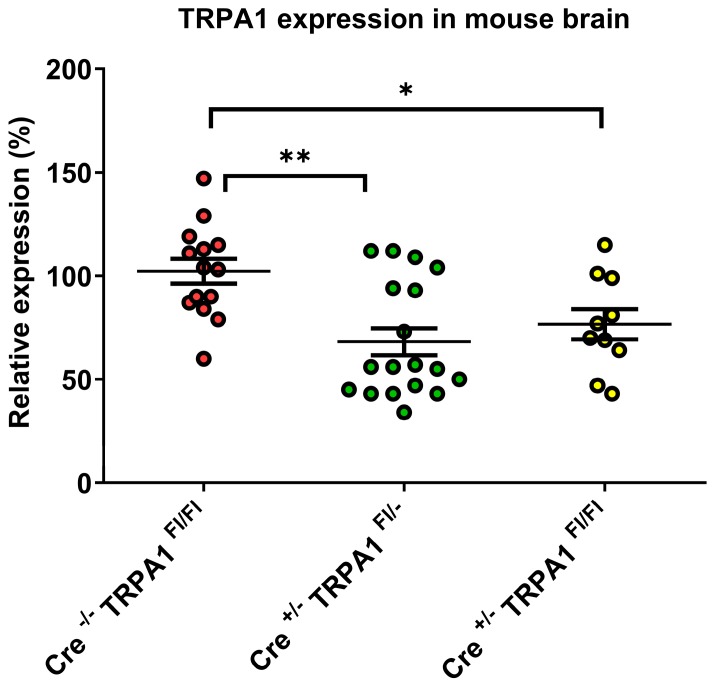
Relative expression levels of TRPA1 mRNA in the brain in GFAP-Cre^−/−^ TRPA1^Fl/Fl^ (*n* = 7), GFAP-Cre^+/−^ TRPA1^Fl/−^ (*n* = 5) and GFAP-Cre^+/−^ TRPA1^Fl/Fl^ mice (*n* = 9). Asterisks show statistically significant differences between the indicated groups (*p < 0.05, **p < 0.01).

Intact TRPA1 mRNA levels were determined by quantitative RT-PCR in brain samples of GFAP-Cre^-/-^ TRPA1^Fl/Fl^ compared to GFAP-Cre^+/-^ TRPA1^Fl/-^ and GFAP-Cre^+/-^ TRPA1^Fl/Fl^ mice. The obtained relative expression levels, means and standard deviations are presented in Figure 1 for each group. Compared to GFAP-Cre^−/−^ control mice where TRPA1 expression level was 102 ± 22%, both the GFAP-Cre^+/−^ TRPA1^Fl/−^ and GFAP-Cre^+/−^ TRPA1^Fl/Fl^ mouse groups had lower expression levels, 68 ± 28% and 77 ± 23%, respectively. These results indicate that some of the TRPA1 mRNA was lost due to Cre-LoxP recombination in both GFAP-Cre^+/−^ groups. ANOVA statistical analysis showed that there was a significant difference between the groups (p=0.0017). The post hoc test demonstrated that the differences were significant when both the GFAP-Cre^+/−^ TRPA1^Fl/−^ and GFAP-Cre^+/−^ TRPA1^Fl/Fl^ group were compared to the GFAP-Cre^−/−^ TRPA1^Fl/Fl^ control group (p < 0.01 and p < 0.05 respectively), but there was no significant difference in TRPA1 mRNA expression between the GFAP-Cre^+/−^ groups (Figure 1).

### 3.2. Magnetic Resonance Imaging (MRI)

**Figure 2:**
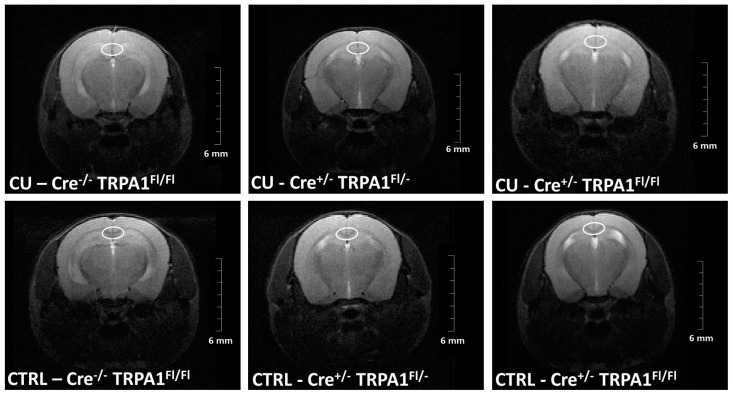
Representative MR images, constructed on fourth week. The medial corpus callosum is highlighted by a white ellipse.

The timeline of damage was followed by the evaluation of signal intensity changes of T2 weighted MRI (Figure 2) in the medial part of the corpus callosum between week 2 and 6.

**Figure 3:**
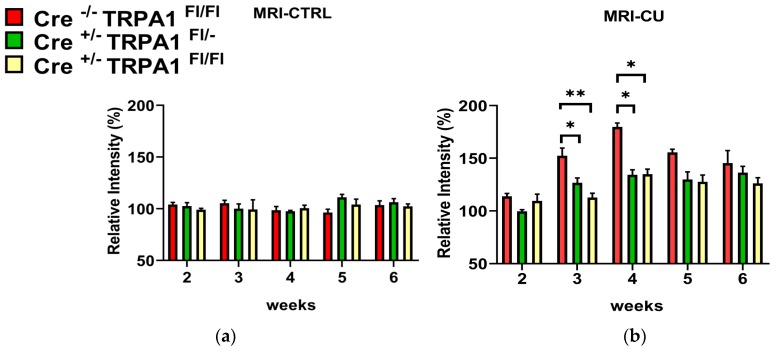
Changes of signal intensity in the medial corpus callosum measured by magnetic resonance imaging between week 2 and 6 of cuprizone treatment in **(a)** control (CTRL) and **(b)** cuprizone-treated (CU) groups of GFAP-Cre^−/−^ TRPA1^Fl/Fl^, GFAP-Cre^+/−^ TRPA1^Fl/−^ and GFAP-Cre^+/−^ TRPA1^Fl/Fl^ mice. Data are means ± S.E.M. of images obtained from animals (n = 4/group). The signal intensities were normalized to the baseline values (100%) for each animal and indicated by percentages of relative intensity. Asterisks show statistically significant differences between respective CU and CTRL groups (* *p* < 0.05, ** *p* < 0.01).

The intensities in the untreated control groups were not significantly different and no changes were detected in any of the control animals throughout the whole experiment. (Figure 3a). In contrast, the signal intensity was significantly increased in cuprizone-treated GFAP-Cre^-/-^ TRPA1^Fl/Fl^ mice compared to GFAP-Cre^+/−^ TRPA1^Fl/−^ and GFAP-Cre^+/−^ TRPA1^Fl/Fl^ animals on week 3 and week 4 (*p < 0.05, **p < 0.01; Figure 3b). The most pronounced increase was detected on the fourth week, 179.75% in GFAP-Cre^−/−^ TRPA1^Fl/Fl^ group versus 134.25% (GFAP-Cre^+/−^ TRPA1^Fl/−^) and 134.75% (GFAP Cre^+/−^ TRPA1^Fl/Fl^). These results indicate that the myelin damage was less severe in the corpus callosum of both heterozygous and homozygous mGFAP-Cre conditional TRPA1 knockout mice on weeks 3 and 4. The difference between genotypes decreased at later time points. At the end of the treatment we did not detect significant differences between the cuprizone-treated GFAP-Cre^−/−^ TRPA1^Fl/Fl^, GFAP-Cre^+/−^ TRPA1^Fl/−^ and GFAP-Cre^+/−^ TRPA1^Fl/Fl^ animals. Each cuprizone-treated group showed a significantly elevated intensity from the third to sixth weeks compared to their respective control groups (Figure 3a,b; significance not shown).

### 3.3. Cuprizone-Induced Demyelination Determined by LFB/CV Staining and MBP Immunohistochemistry

**Figure 4:**
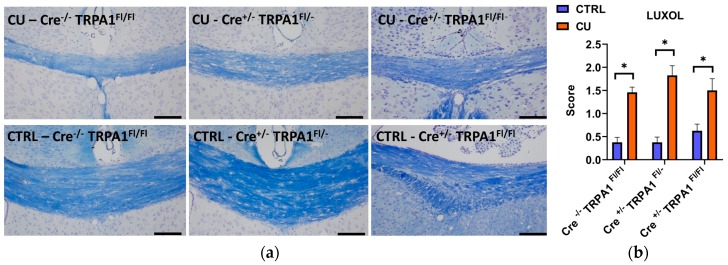
Evaluation of myelin content with Luxol Fast Blue/cresyl violet (LFB/CV) staining after 6 weeks of cuprizone treatment **(a).** Representative images of control (CTRL) and cuprizone-treated (CU) groups in GFAP-Cre^−/−^ TRPA1^Fl/Fl^, GFAP-Cre^+/−^ TRPA1^Fl/−^ and GFAP-Cre^+/−^ TRPA1^Fl/Fl^ mice.; **(b)** Semiquantitative demyelination scores of LFB/CV staining, *p < 0.05, n = 3–5/group, Scale bar = 100 µm.

**Figure 5:**
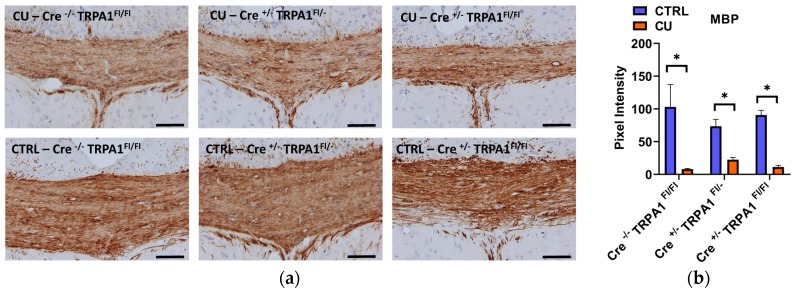
Evaluation of myelin content with myelin basic protein (MBP) immunohistochemistry after 6 weeks of cuprizone treatment **(a).** Representative images of control (CTRL) and cuprizone-treated (CU) groups in GFAP-Cre^−/−^ TRPA1^Fl/Fl^, GFAP-Cre^+/−^ TRPA1^Fl/−^ and GFAP-Cre^+/−^ TRPA1^Fl/Fl^ mice.; **(b)** Pixel intensities of diaminobenzidine staining in the middle part of the corpus callosum in each group, *p < 0.05, n = 3–5/group, Scale bar = 100 µm.

After the six weeks of the treatment, significant demyelination was detected in all three cuprizone-treated groups with the LFB/CV staining, compared to their respective control groups (Figure 4a,b). No statistically significant differences were found between the demyelination scores of the three genotypes. Likewise, immunohistochemical staining of MBP revealed a significant reduction in all cuprizone-fed animals compared to controls, but no difference was detected between the MBP content of GFAP-Cre^−/−^ TRPA1^Fl/Fl^, GFAP-Cre^+/−^ TRPA1^Fl/−^ and GFAP-Cre^+/−^ TRPA1^Fl/Fl^ mice (Figure 5a,b). The results show that the attenuating effect of mGFAP-driven conditional deletion of the TRPA1 receptor on the severity of cuprizone-induced demyelination was diminished by week 6 of treatment.

### 3.4. Cuprizone-Induced Astrocyte and Microglia Activation

**Figure 6:**
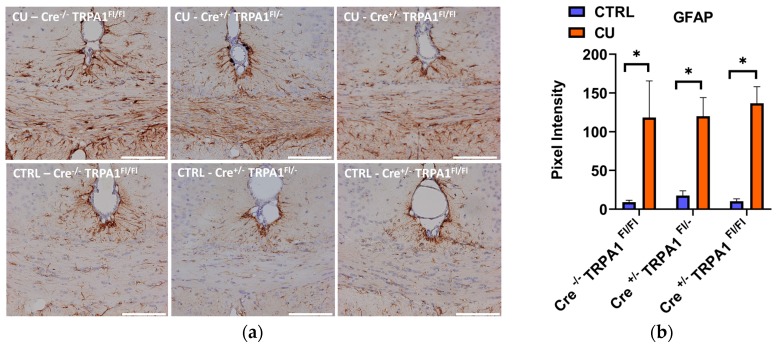
Evaluation of astrocyte activation by GFAP immunohistochemistry after 6 weeks of cuprizone treatment **(a)** Representative images of control (CTRL) and cuprizone-treated (CU) groups in GFAP-Cre^−/−^ TRPA1^Fl/Fl^, GFAP-Cre^+/−^ TRPA1^Fl/−^ and GFAP-Cre^+/−^ TRPA1^Fl/Fl^ mice.; **(b)** Pixel intensities of diaminobenzidine staining in the middle part of the corpus callosum in each group; *p < 0.05, n = 3–5/group, Scale bar = 100 µm.

**Figure 7:**
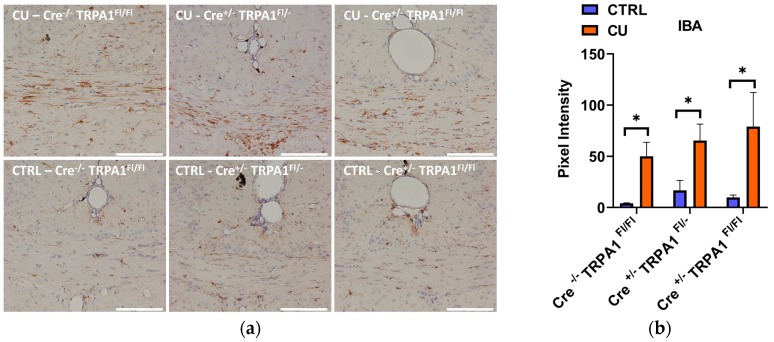
Evaluation of microglia activation by Iba1 immunohistochemistry after 6 weeks of cuprizone treatment **(a)** Representative images of control (CTRL) and cuprizone-treated (CU) groups in GFAP-Cre^−/−^ TRPA1^Fl/Fl^, GFAP-Cre^+/−^ TRPA1^Fl/−^ and GFAP-Cre^+/−^ TRPA1^Fl/Fl^ mice.; **(b)** Pixel intensities of diaminobenzidine staining in the middle part of the corpus callosum in each group *p < 0.05, *n* = 3–5/group, Scale bar = 100 µm.

At the end of the 6-week treatment, a significant increase of GFAP and Iba1 immunostaining was detected in all three cuprizone-treated groups, compared to their respective control groups (Figure 6a and Figure 7a). Quantifying the intensity of the staining, no statistically significant differences were found in the DAB staining intensities between the three genotypes (Figure 6b and Figure 7b). Therefore, by the end of the 6-week treatment the mGFAP-driven conditional deletion of the TRPA1 receptor had no effect on the astrocyte and microglia accumulation induced by cuprizone.

## 4. Discussion

In the present study we have investigated the modulatory role of TRPA1 receptors expressed by GFAP positive cells in the cuprizone-induced demyelination model using mGFAP-driven conditional TRPA1 receptor knockout mice. The glial fibrillary acidic protein (GFAP) is a generally accepted marker of astrocytes. Brain diseases are characterized by the active inflammatory state of astrocytes, which is usually manifested as up-regulation of GFAP [28].

Our results show that the genetic lack of TRPA1 receptors in GFAP positive cells influences the demyelination process induced by cuprizone feeding in mice. Reduced pathological changes were detected in the corpus callosum by MRI between the 3rd and 5th weeks of the treatment. In contrast, at the end of week 6, no significant differences were found in the demyelination evaluated either by MRI or histology.

In a previous study using embryonic global TRPA1 KO animals we demonstrated that TRPA1 receptor deficiency attenuated the cuprizone-induced demyelination by reduction of apoptosis of mature oligodendrocytes [22]. Additionally, it was recently published that oligodendrocytes express TRPA1 receptors [11]. Therefore, a direct effect of TRPA1 activation on oligodendrocyte apoptosis in the cuprizone model could be hypothesized. Since our investigations have not provided sufficient evidence for TRPA1 expression in oligodendrocytes, it was presumed that TRPA1 receptor deficiency might alleviate the cuprizone-induced loss of mature oligodendrocytes by altering the release of mediators by astrocytes. TRPA1 receptors localized on astrocytes may participate in the astrocyte-oligodendrocyte crosstalk. Since TRPA1 can be triggered by several noxious stimuli, including inflammation, tissue damage or oxidative stress on one hand, and astrocytes influence the extent of this toxin-induced demyelination on the other [29], we assumed that this receptor might modulate the demyelination process. Numerous earlier studies have provided substantial morphological and functional evidence that astrocytes express the TRPA1 receptor, functioning as a regulator of resting Ca^2+^ levels [8,9,24,30,31]. In a very recent study, Oh et al., using the cell-type-specific gene-silencing and ultrasensitive sniffer-patch techniques, identified astrocytes as the cellular target and TRPA1 as the molecular sensor for the low intensity, low frequency ultrasound (LILFU). They demonstrated that LILFU-induced neuromodulation was initiated by opening of TRPA1 channels in astrocytes. The Ca^2+^ entry through TRPA1 caused a release of gliotransmitters including glutamate from the astrocytes activating NMDA receptors in neighboring neurons [32]. In another newly-published paper, Xia et al. raised the involvement of TRPA1 in myelin damage and oxidative stress injury in a mouse intracerebral hemorrhage (ICH) model. They showed that TRPA1 was activated by the increased reactive oxygen species (ROS) after ICH, leading to an increase of Ca^2+^ influx. The increased Ca^2+^ further contributed to the rise in NOX1 and Calpain1, causing oxidative stress damage and myelin degradation. [33].

Since our previous data also supported the TRPA1 immunopositivity of astrocytes in the corpus callosum and astrocytic reactions were less prominent in cuprizone-treated TRPA1 receptor deficient mice compared to wild-type counterparts, we have supposed a pivotal role of TRPA1 receptors expressed by astrocytes [22]. For our further investigations, mGFAP-driven conditional TRPA1 knockout mice were bred by our laboratory in order to reveal the precise role of astrocytic TRPA1 receptors. These mice were created via crossbreeding floxed TRPA1 carrying mice (B6.129S-Trpa1tm2Kykw/J) by GFAP promoter-directed Cre recombinase gene expressing mice (B6.Cg-Tg(Gfap-cre) 77.6Mvs/2J. This technique allows the selective cutout of the Trpa1 gene only from the GFAP-positive cells and the examination of cuprizone-induced demyelination in the lack of astrocyte-specific TRPA1 receptors.

Cuprizone-treatment significantly increased T2-weighted MRI signal intensities measured on the 3rd and 4th weeks in all three animal groups compared to the baseline, proving the reliability of the model. Myelin loss with the concomitant water accumulation enhances the signal intensity on T2-weighted MR images [34]. The intensity of T2-weighted images of ROIs corresponding to the medial corpus callosum was reproducible. Remarkably, the MRI was sensitive enough to notice the milder severity of myelin loss in GFAP-Cre^+/−^ TRPA1^Fl/−^ and GFAP-Cre^+/−^ TRPA1^Fl/Fl^ animals compared to the GFAP-Cre^−/−^ TRPA1^Fl/Fl^ group. The most pronounced difference was detected on the 4th week, but the disparity decreased at later time points. In our previous study in the cuprizone model with embryonic global TRPA1 KO mice, we provided MRI-based evidence that the severity of myelin loss in the gene-deleted animals was significantly lower at all measurement points during the six-week experiment compared to the wild-type controls [23]. In accordance with the present results, the largest differences in signal intensities were detected on the 3rd and 4th weeks. Considering the data of the follow-up in vivo imaging, we conclude that genetic loss of the astrocyte-specific TRPA1 receptors attenuates the progress of demyelination in the corpus callosum, but these effects can be noticed only at the time of the most intensive pathological changes. The homozygote (Cre^+/−^ TRPA1^Fl/Fl^) and heterozygote (Cre^+/−^ TRPA1^Fl/−^) mice showed similar alterations which is explained by a similar reduction of mRNA levels in both genotypes. However, by the 6th week these disparities had disappeared, as we could not detect any differences with histological examinations at the end of the study. Neither the semiquantitative scoring of demyelination, based on Luxol fast blue staining technique, nor the MBP immunohistochemistry showed significant differences in the mGFAP-driven conditional TRPA1 knockout mice compared to the Cre^-/-^ controls. Similarly to the demyelination, the cuprizone-induced accumulation of the astrocytes and microglial cells in the corpus callosum was not influenced by the genetic loss of TRPA1 in the GFAP positive cells. In contrast to the results obtained with embryonic global TRPA1 KO mice, these results suggest that deletion of the receptor in the astrocytes does not exert substantial inhibitory effect eventually on the six-week cuprizone treatment-induced demyelination of the corpus callosum.

## 5. Conclusions

According to the currently available data, we presume that TRPA1 receptors localized on astrocytes can be activated by electrophilic ligands, reactive oxygen species in response to cuprizone challenge and the consequent release of pro-inflammatory mediators contributes to the progression of oligodendrocyte apoptosis. However, since the global TRPA1 KO mice presented a more attenuated demyelination compared to the conditional KO mice, it can be concluded that TRPA1 receptors on astrocytes contribute to the demyelination induced by cuprizone only transiently and TRPA1 receptors expressed by other cell types in the brain (e. g. oligodendrocytes, microglia, neurons) may participate in the demyelination process [11,33]. The expression and function of TRPA1 receptors by other cells in the brain warrant further investigation.
